# Occurrence of papillary renal cell carcinoma and clear cell renal carcinoma in a patient: A unique case report

**DOI:** 10.1097/MD.0000000000042312

**Published:** 2025-05-02

**Authors:** Zdravka Harizanova, Ferihan Popova, Vasil Pavlov, Elena Bozhikova

**Affiliations:** a Department of Anatomy, Histology and Embryology, Faculty of Medicine, Medical University – Plovdiv, Plovdiv, Bulgaria; b Urology Department, University Hospital for Active Treatment “Kaspela,” Medical University – Plovdiv, Plovdiv, Bulgaria; c Department of Biomedical Sciences, Mercer University School of Medicine, Columbus, GA.

**Keywords:** clear cell renal cell carcinoma, inheritance, kidney, papillary renal cell carcinoma

## Abstract

**Rationale:**

Renal cancer is one of the most common neoplasms in both males and females. Precise diagnosis, grading, and staging are very important for the outcome and the prognosis of the malignant process. Renal carcinoma disorders are presented by kidney tumors usually of the same histological type. The presence of various tumor histological types is an extremely rare event.

**Patient concerns:**

Two different histological types of tumors were found in the left kidney of a 74-year-old man.

**Diagnoses:**

The diagnosis obtained was papillary renal cell carcinoma type 1 and clear cell renal carcinoma with pathologic stage T2N0M1.

**Interventions and outcomes:**

After abdominal ultrasound and computer tomography, consultation with an anesthesiologist, and a cardiologist, the patient underwent radical left nephrectomy.

**Lessons:**

Pathologists must be aware of the possibility of the presence of more than one histological type of renal carcinoma due to genomic alterations. Further genetic investigations must be conducted to identify the specific type and thus the treatment will be most precise.

## 1. Introduction

Renal cancer is one of the most common neoplasms in both males and females.^[1]^ Surgical removal is gold standard treatment for localized renal tumors while for the treatment of metastatic renal cell carcinoma targeted therapies have been conducted.^[2]^ Precise diagnosis, grading and staging are very important for the outcome and the prognosis of the malignant process. Tumor size, perinephric fat extension, renal sinus invasion, lymph node, renal vein and vena cava, adrenal gland involvement are significant determinants of the pathologic stage. Papillary renal cell carcinoma (pRCC) constitutes 7% to 15% of renal cell carcinoma, thus being the second most common type after clear cell type. It is a histological subtype which differs from the clear cell type cytogenetically and it even has different prognosis. Papillary renal cell carcinoma in turn has 2 morphological subtypes: subtype 1 and subtype 2 with a worse prognosis. It is more common in males, patients with acquired kidney cysts and patients undergoing dialysis.^[3]^ It develops as a single tumor or multifocal and bilateral one. Recently, the International Society of Urological Pathology (ISUP) recognizes clear cell papillary renal cell carcinoma as a distinct epithelial tumor exhibiting characteristics of both pRCC and clear cell renal cell carcinoma (ccRCC) but distinguished from them by genetic differences in the von Hippel-Lindau (VHL) tumor suppressor gene mutation.^[4]^

It is considered that 3% of renal carcinomas are associated with inherited predisposition.^[5]^ Therefore, many genes involved in autosomal dominant syndromes have been identified among which VHL tumor suppressor gene is most frequent. Its germline mutation refers to the occurrence of ccRCC.^[6]^ On the other hand, hereditary pRCC is a very rare malignancy, characterized by multifocal and bilateral papillary type 1 pRCC and papillary adenomas. However, familial renal cell carcinoma (RCC) disorders are presented by bilateral and multifocal kidney tumors usually of the same histological type. Presence of various histological tumors in 1 patient is an extremely rare event.

We reported a case of a patient with both pRCC and ccRCC in the left kidney which is a rare condition of 2 different histological types of tumors in 1 kidney at the same time.

## 2. Case report

A 74-year-old man underwent an abdominal ultrasound which showed a right kidney with normal topic, size and parenchyma, no drainage disorders and a left kidney with normal topic, size and no drainage disorders with a heterogenous formation with size 82/60 mm on the superior extremity. Subsequent native and contrast computed tomography in arterial and venous phase revealed solid mass with smooth and distinct borders with 31 Hounsfield units (HU) density and axial size 8.5/6.6 cm. involving the superior pole of the left kidney. The formation impounded up to 41 HU in the arterial phase and to 58 HU in the venous phase. A similar lesion in the middle part of the renal parenchyma was found with size 1.4 cm and density 85 HU in the venous phase. There was moderate fibrosis in both lungs. The liver had normal size and density, an oval hypodense formation in the left lobe with size 1.7 cm. was found and similar mass in the right lobe was presented. The gall bladder had normal size and density, no intra- or extrahepatic cholestasis. Adrenal glands and urinary bladder were also normal, no enlarged lymph nodes. The patient had no familial background of RCC. Laboratory tests were remarkable for a slightly elevated white blood cell count of 11.39 × 10^3^ cells/mm^3^ (normal range 4.5–11.00 × 10^3^), the red blood cells, hemoglobin, hematocrit, mean corpuscular hemoglobin, concentration, and volume were normal. Urinalysis revealed urine with a normal specific gravity of 1.020, 2 + protein, no glucose, ketones, bilirubin, urobilinogen and 1 white and 1 red blood cell.

A week later after consultation with an anesthesiologist and a cardiologist the patient underwent radical left nephrectomy. He was observed in the hospital for 6 days and was discharged home. The man visited the hospital 10 days after this for a checkup. He was directed to further immunohistochemical investigation for definitive diagnosis and oncology committee for further complex treatment.

## 3. Pathological findings

**Figure 1.**
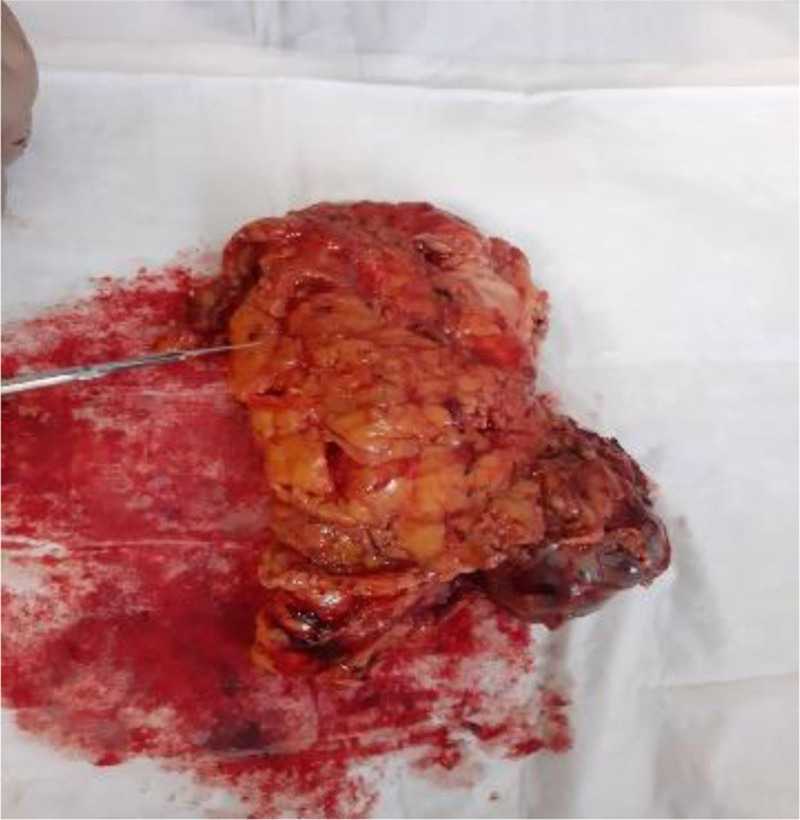
Nephrectomy specimen.

**Figure 2.**
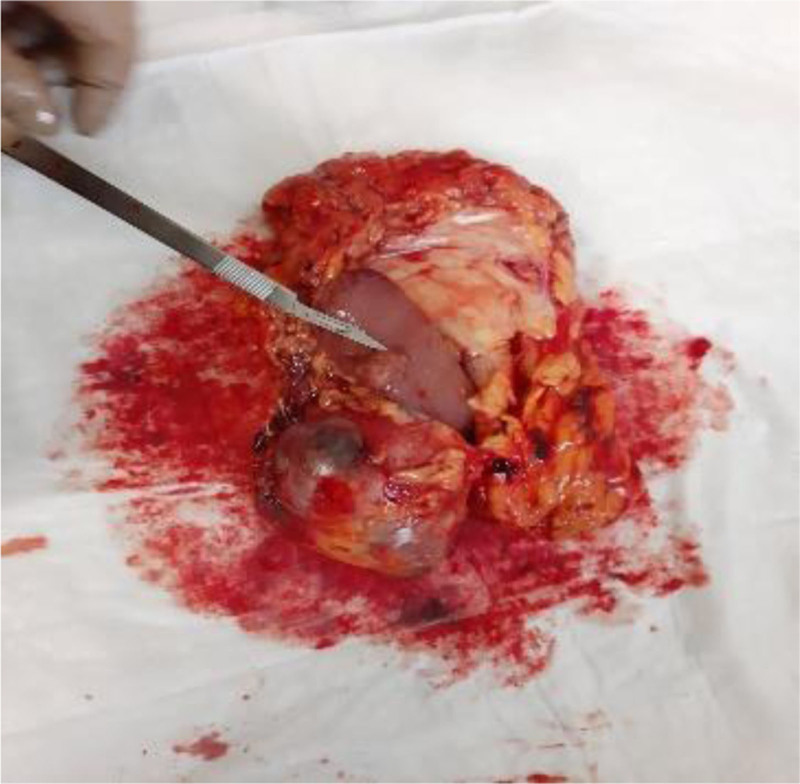
Large heterogenous and necrotic mass.

**Figure 3.**
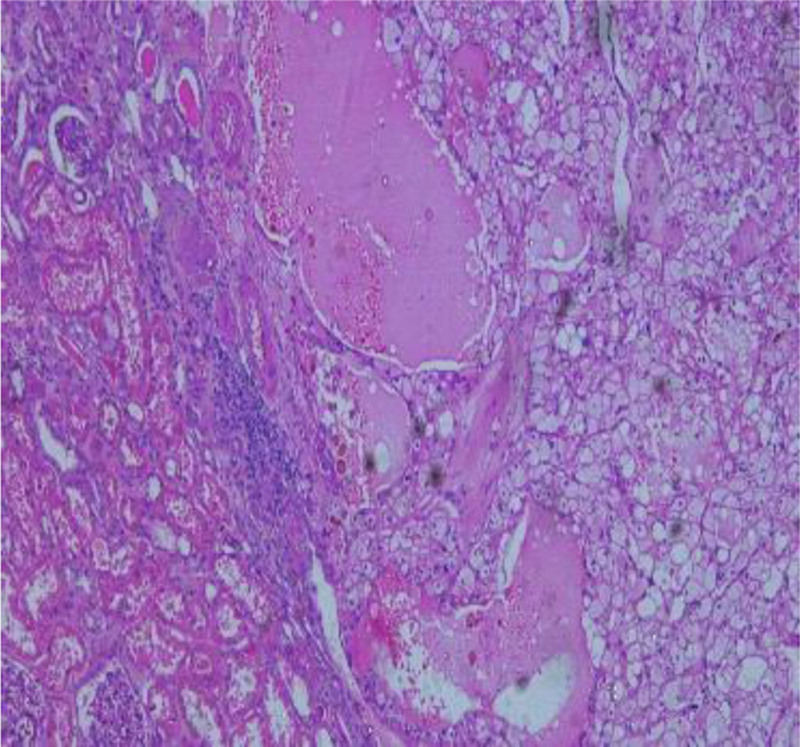
pRCC type I characterized by papillary cores covered by a single layer of tumor cells (magnification 100×). pRCC = papillary renal cell carcinoma.

**Figure 4.**
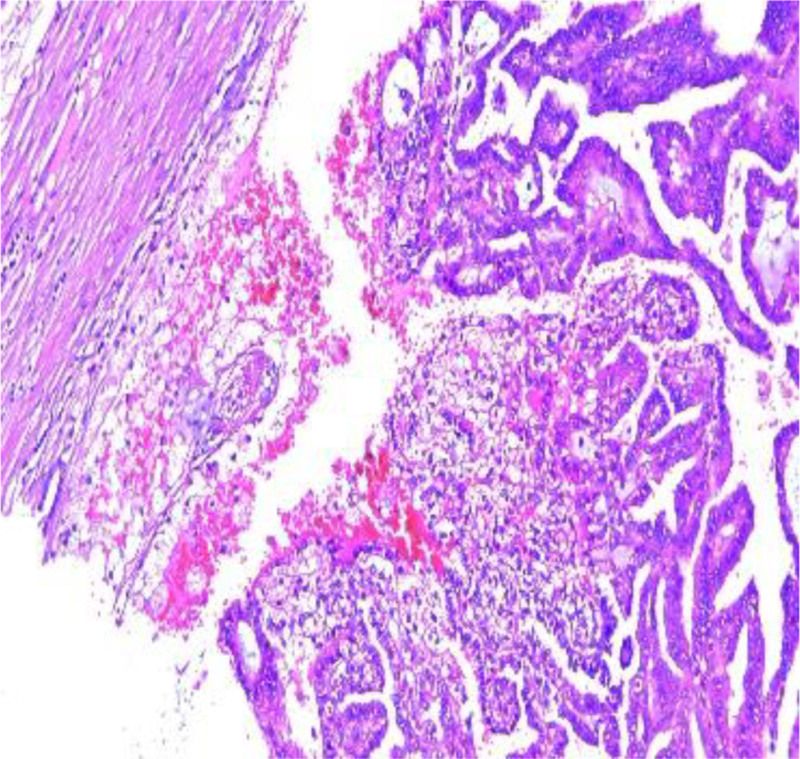
Renal fibrous capsule invasion (magnification 100×).

**Figure 5.**
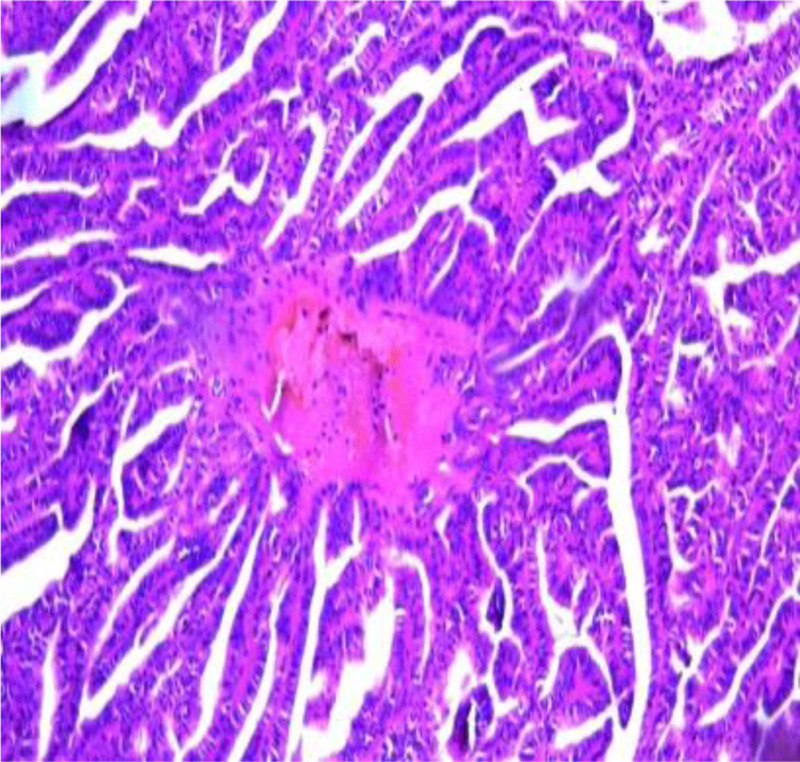
ccRCC with blood-filled microscopic cysts (magnification 100×). ccRCC = clear cell renal cell carcinoma.

**Figure 6.**
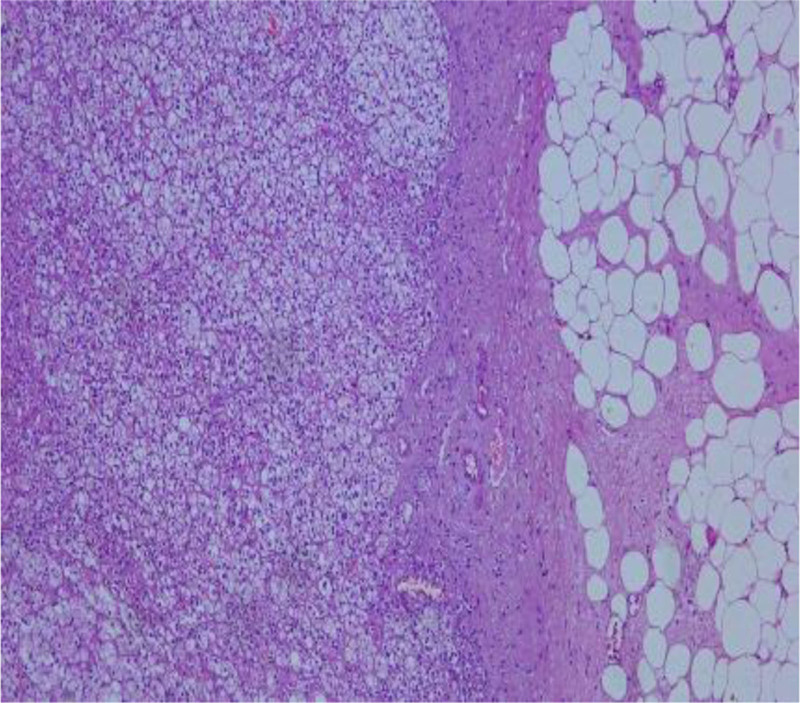
Adrenal hyperemia (magnification 100×).

The left kidney, adrenal gland and the renal fat capsule were removed (Fig. 1). A necrotic hemorrhagic mass was identified on the upper pole of the specimen (Fig. 2) measured 8 × 7 × 5 cm. The tumor had a friable and heterogeneous surface with firm areas. The whole specimen was sent for histopathologic investigation. The diagnosis obtained was papillary renal cell carcinoma type 1 according to ISUP grade 3 with renal fibrous capsule invasion (Figs. 3 and 4). The tumor exhibited areas of stellate tubular structures showing ribbon-like layering configuration around hyalinized zones. A second tumor formation centered within the middle of the renal parenchyma was found which was classified as clear cell renal cell carcinoma with diameter 1.5 cm, again according to ISUP grade 3 and involvement of the renal capsule (Fig. 5). Adrenal hyperemia was found (Fig. 6). The histopathological features of our case are presented in Figures 3–6. The first tumor (pRCC) had papillary and tubular architecture, with nests of large eosinophilic cells surrounded by smaller amphophilic cells forming alveolar patterns (100× magnification). Infiltration of foamy macrophages was observed. The second tumor had the characteristics of clear cell renal carcinoma with blood-filled microscopic cysts. No invasion of the perirenal fat. Final pathologic stage of T2N0M1 was defined.

An ethical approval was taken for this publication by the Ethics committee in Medical University-Plovdiv. An informed consent was signed by the patient.

The datasets generated and analyzed during the current study are available from the corresponding author on reasonable request.

## 4. Discussion

The presented case of combined presence of clear cell renal cell carcinoma and papillary cell renal carcinoma type 1 should be considered as casuistic. The occurrence of synchronous kidney tumors of different histological subtypes in a patient is described also in a 51-year-old man whom genetic analysis showed germline mutation in the MET proto-oncogene.^[6]^ It is located on chromosome 7q31 and encodes the receptor for hepatocyte growth factor. Traditionally, a specific histological type is described according to each inherited syndrome: clear cell renal cell carcinoma in VHL disease, papillary tumors in HPRC C syndrome. Other cases of occurrence of mixed clear cell renal cell carcinoma with type 1 papillary carcinoma, have been reported also.^[7–9]^ Several hypotheses could be considered to explain the various histological types: common metabolic pathway, specific type of mutation, common origin—both ccRCC and pRCC originate from the proximal tubules.^[10]^ Additional genetic events of key regulatory genes that push the tumor to a particular phenotype also should be considered. Recently, a case of a 73-year-old woman with presence of papillary cell carcinoma and malakoplakia in the same kidney which was the third of this kind described had been reported.^[11–13]^ Renal malakoplakia can imitate renal cell carcinoma.^[14]^ The fact that different histological types of malignant renal disorders can occur has diagnostic and therapeutic significance. Pathologists are challenged to reach the correct diagnosis and should be alerted to the fact that pRCC can occur alongside ccRCC or malakoplakia and that the diagnosis of one does not exclude the other. The precise diagnosis is of great significance because surgical options are more appropriate in pRCC cases but if it is combined with other histological type, treatment with reduced immunosuppression or a combination of cholinergic agonists (bethanechol chloride) and antibiotics should also be considered.^[15,16]^

Our case also reveals the potential misdiagnosis on small biopsy. A small sample may contain pRCC and thus result in missed diagnosis of ccRCC. To ensure the detection of all histological types, careful attention by the pathologist to separate and examine many areas of the specimen is needed. Accurate histological staging and grading of the renal cell carcinoma is also of great significance. Now guidelines produced by the ISUP are used for classification and grading of the renal tumors.^[2]^ According to the ISUP Grading Classification there are 4 grades in the renal carcinoma staging. Grades 1 to 3 are based on nucleolar prominence. Grade 1 indicates invisible or small and basophilic at 400× magnification tumor cell nucleoli, grade 2 exhibits conspicuous at 400× magnification but inconspicuous at 100× magnification tumor cell nucleoli, in grade 3 tumor cell nucleoli are eosinophilic and clearly visible at 100× magnification. Grade 4 is defined as tumors with highly pleomorphic tumor giant cells or the presence of sarcomatoid and rhabdoid morphology.^[17]^ This classification refers to pRCC and ccRCC. Both tumors are grade 3 in the presented case. Additional prognostic factors are also important such as tumor necrosis and tumor morphotype.^[18]^ It is noted that ccRCC has a worse outcome than chromophobe or pRCC. Subtyping of pRCC into 1 and 2 also provides prognostic information with subtype 2 having worse prognosis than 1.^[19]^

## 5. Conclusion

Pathologists must be aware of the possibility of presence of more than 1 histological type of renal carcinoma due to genomic alterations which will help avoid the misdiagnosis of certain types of renal cell carcinoma. Further genetic investigations must be conducted to identify the specific type and thus the treatment will be most precise.

## Author contributions

**Conceptualization:** Zdravka Harizanova, Ferihan Popova, Vasil Pavlov, Elena Bozhikova.

**Investigation:** Zdravka Harizanova, Vasil Pavlov.

**Supervision:** Zdravka Harizanova, Ferihan Popova, Vasil Pavlov.

**Visualization:** Elena Bozhikova.

**Writing – original draft:** Zdravka Harizanova.

**Writing – review & editing:** Ferihan Popova, Elena Bozhikova.
